# Genetic communication by extracellular vesicles is an important mechanism underlying stem cell-based therapy-mediated protection against acute kidney injury

**DOI:** 10.1186/s13287-019-1227-8

**Published:** 2019-04-17

**Authors:** Lingfei Zhao, Chenxia Hu, Ping Zhang, Hua Jiang, Jianghua Chen

**Affiliations:** 1 Kidney Disease Center, the First Affiliated Hospital, College of Medicine, Zhejiang University, Hangzhou, Zhejiang Province People’s Republic of China; 2 Key Laboratory of Kidney Disease Prevention and Control Technology, Hangzhou, Zhejiang Province People’s Republic of China; 3 Institute of Nephrology, Zhejiang University, Hangzhou, Zhejiang People’s Republic of China; 4 State Key Laboratory for Diagnosis and Treatment of Infectious Diseases, the First Affiliated Hospital, College of Medicine, Zhejiang University, Hangzhou, Zhejiang People’s Republic of China

**Keywords:** Stem cell-based therapy, Extracellular vesicles, Genetic communication, Acute kidney injury

## Abstract

Stem cell-based therapy appears to be a promising new candidate for acute kidney injury (AKI) management. Traditionally, it has been accepted that the mechanism underlying the regenerative effect of stem cells is based on their paracrine/endocrine activity, including release of bioactive factors that act on injured renal cells and presentation of proangiogenic, antiapoptotic, antioxidative, and immunomodulatory effects. Recently, multiple studies have confirmed that extracellular vesicles (EVs) are a kind of vesicle rich in a broad variety of biologically active molecules, including lipids, proteins, and, in particular, nucleic acids. EVs are able to transfer genetic information to target cells, alter target gene regulatory networks, and exert biological effects. Stem cell-derived EVs (SC-EVs) are emerging as potent genetic information sources that deliver mRNAs and miRNAs to injured renal cells and exert renoprotective effects during AKI. On the other hand, EVs originating from injured renal cells also contain genetic information that is believed to be able to influence phenotypic and functional changes in stem cells, favoring renal recovery. In this review, we summarize studies providing evidence of genetic communication during the application of stem cells in preclinical AKI models, aiming to clarify the mechanism and describe the therapeutic effects of stem cell-based therapy in AKI patients.

## Background

During recent decades, stem cell-based therapy has emerged as a promising candidate for acute kidney injury (AKI) management. While pharmacologic interventions often target only one single aspect of the highly complex pathophysiological processes that occur after AKI, stem cell-based therapy may have the advantage of acting through multiple mechanisms to promote renal repair and recovery [1]. Considerable preclinical evidence has confirmed the effectiveness of stem cell-based therapy in improving the prognosis of AKI in different animal models, including models of ischemia/reperfusion (I/R) injury, cisplatin-induced injury, glycerol-induced injury, and sepsis, which represent most causes of human AKI [2–5]. The main mechanism underlying the protective effects of stem cell-based therapy is commonly regarded to be its paracrine/endocrine activity, including release of bioactive factors that act on target cells and presentation of proangiogenic, antiapoptotic, antioxidative, and immunomodulatory effects [6–9]. Despite all the promising preclinical results, there still exist some concerns regarding the clinical application of stem cell-based therapy in AKI, especially with regards to vascular occlusion, tumorigenicity, and immunoreactivity [10]. Research on safer and more effective strategies for stem cell-based therapy are urgently needed.

Extracellular vesicles (EVs) are a heterogeneous population of vesicles that appear to be released by most cell types, including stem cells, into the extracellular environment. EVs are rich in a broad variety of biologically active molecules, including lipids, proteins, and nucleic acids (e.g., mRNAs, noncoding RNAs, and miRNAs) [11]. Depending on their cytoplasmic constituents, EVs can play important roles in the regulation of cell function and tissue regeneration through cell–cell communication [12]. It has been reported that EVs released from stem cells may transfer genetic information to injured renal cells and mimic the beneficial effects of stem cell-based therapy in the treatment of AKI [13]. Because it avoids the possibility of immune rejection, vascular occlusion, and tumor generation, this cell-free strategy may become a promising therapeutic approach for AKI management.

In this review, by summarizing previous studies, we provide an integrated and up-to-date view of the role of genetic communication by EVs between stem cells and injured renal cells in the process of protection against acute kidney injury, with the aim of clarifying the mechanism and describing the therapeutic effects of stem cell-based therapy in AKI patients.

## Characteristics of EVs

The first article demonstrating the existence of EVs was published by Johnstone et al. in 1983. The authors described the existence of vesicles that contained peptides and were released during the incubation of sheep reticulocytes [14]. Since then, different types of EVs, together with their different biogenesis pathways and biophysical properties, have been very widely reported. Although there is no consensus on their classification, EVs can generally be classified into three main categories: exosomes, microvesicles (MVs), and apoptotic bodies. Exosomes originate from the endocytic recycling pathway. For this reason, they tend to be a rather homogenous population of small particles. Exosomes share some common characteristics not only with regards to size (40–100 nm) but also with regards to some markers, such as their expression of tetraspanins (CD9, CD63, and CD81), their low amounts of phosphatidylserine, and their enrichment for heat shock proteins (HSPs) (HSP60, HSP70, and HSP90), apoptosis-linked gene-2-interacting protein x (Alix), clathrin, and annexin [15]. After fusion of multivesicular bodies with the plasma membrane, exosomes are subsequently released into the extracellular space in a manner dependent on cytoskeletal activation [16]. On the other hand, MVs are a relatively heterogeneous population of vesicles, with sizes ranging from 100 nm to 1 μm [17]. They are released by direct budding from the plasma membrane, which is dependent on calcium influx and cytoskeletal activation [18]. Due to their formation mechanism, they share similar surface markers with their parent cells [19] and are rich in cholesterol, phosphatidylserine, and flotillin-1 [20, 21]. A third type of EV includes apoptotic bodies, which have sizes larger than 1 μm. Apoptotic bodies are derived from fragments of the plasma membrane and are the only type of EVs with DNA [22].

## EVs participate in various physiological and pathological processes through cell-to-cell communication

**Fig. 1:**
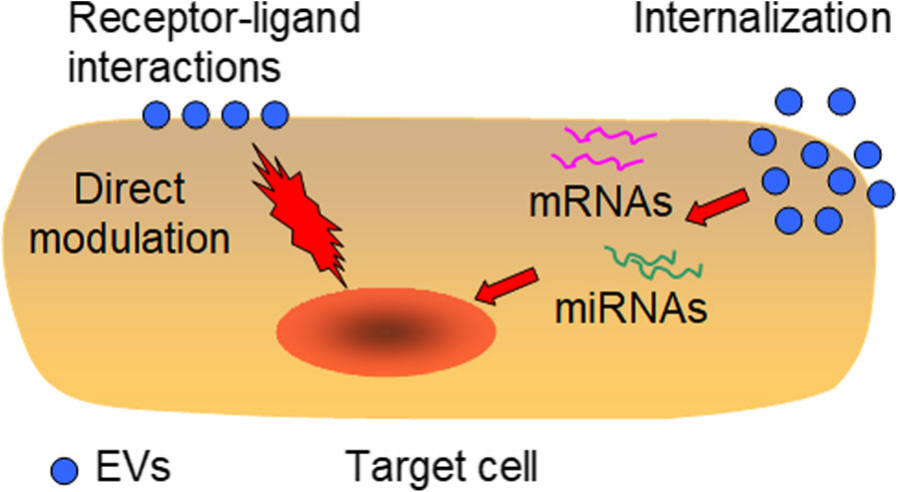
Mechanisms by which EVs influence the behavior of recipient cells. EVs can either directly modulate the functions of target cells through receptor-ligand interactions or fuse with target cells and modify their activity by delivering mRNAs and miRNAs after internalization. EVs, extracellular vesicles

EVs express a series of unique surface molecules and contain specific cargoes depending on their originating cells. These features have helped them emerge as important mediators of cell–cell communication in both physiological and pathological processes. EVs have the capacity to influence the behavior of recipient cells by several different mechanisms (Fig. 1). They can directly modulate the function of target cells through receptor-ligand interactions [23]. Alternatively, they can fuse with target cells and modify their activity by delivering intracellular cargoes after internalization [24].

Antigenic peptides can be wrapped in EVs, which can participate in the process of antigen presentation, subsequently activate the immune system, and accelerate clearance of various pathogens. Antigen-presenting cells (APCs) secrete exosomes that highly express major histocompatibility complex (MHC) and costimulatory molecules, both of which are essential for T cell activation and proliferation [23, 25]. Some certain microbial components, such as mycobacterial lipids, can also be present in EVs. EVs containing mycobacterial Ag promote effector T cell immunity, serving as pathogen immune surveillance cells [26]. Tumor-derived EVs, which can be taken up by dendritic cells, are also important for stimulating tumor-specific T cell-dependent antitumor effects [27].

Furthermore, EVs are an important kind of immunological component in breast milk. It has been demonstrated that breast milk-derived EVs participate in the development of the infant immune system through cell–cell communication. Breast milk-derived EVs contained large amounts of miRNAs. These miRNAs show acid stability in harsh conditions, indicating that they may be absorbed by infants through the digestive tract. After absorption, these miRNAs can posttranscriptionally modulate various target genes and might be key factors for the development of the infant immune system [28, 29].

In addition to exerting the abovementioned beneficial effects through cell–cell communication, EVs can also be important vehicles for the spread of harmful information. HIV can use EVs as transmission machinery after fusion with target cells through a so-called Trojan horse mechanism [30]. EVs containing prion protein from prion-infected cells have been found to be responsible for the dissemination of this toxic protein to uninfected recipient cells [31]. In the cases of Alzheimer’s disease and Parkinson’s disease, transport of toxic inclusions to other parts of the brain in association with EVs has also been identified [32, 33].

Tumor cells have also been found to secrete large amounts of EVs that communicate with other types of cells, creating a suitable microenvironment for tumor cell survival and metastasis. Exosomes from melanoma cells and ovarian tumor cells contain FasL, which can induce T cell apoptosis and favor tumor cell escape from immune surveillance [34]. It has also been suggested that apoptosis-related proteins and chemotherapeutic drugs could be extruded from tumor cells by EVs [35, 36]. In the context of metastasis, EVs carry multiple active matrix metalloproteinases and proangiogenic signals, which contribute to the invasion and growth of tumor cells [37, 38].

Stem cell-derived EVs (SC-EVs) are a unique population of vesicles released by stem cells that carry both membranes and cytoplasmic contents. Given the natural properties of stem cells, it is not surprising that SC-EVs have emerged as potent genetic information transfer agents that mediate the multiple biological effects of stem cells. The idea that SC-EVs act as cell-cell genetic communication mediators was first proposed by Sharkis et al. The researchers demonstrated that a coculture microenvironment, rather than fusion, was responsible for the conversion of stem cells into cells with liver-specific phenotypes and functions in vitro [39]. Transfer of mRNA by SC-EVs to reprogram neighboring cells was discovered by Ratajczak et al. in 2006 [40]. The delivery of miRNA was also confirmed in 2007 [12]. SC-EVs can act as vehicles that transfer genetic information from donor cells to recipient cells, leading to phenotype switching of recipient cells.

## The role of SC-EVs in AKI

The roles of SC-EVs in the pathophysiologic processes of AKI have received much attention during the last decade. Since Bruno et al. first proved the beneficial effects of SC-EVs in AKI in 2009 [13], many studies have been conducted to verify this result and explore related mechanisms in various AKI models. Although the mechanism of recovery after AKI has not been completely elucidated, it is known that renal cells are not merely passive victims of injury; rather, they actively participate in the repair process [41]. Complete repair after AKI depends on the proliferation and dedifferentiation of surviving renal cells rather than on a source of exogenous progenitor cells [42, 43]. Regarding the mechanism of stem cell-based therapy in AKI, it has also been widely accepted that the regenerative effect might be mediated predominantly by the paracrine action of transplanted stem cells rather than by their ability to directly differentiate into target cells [44, 45]. The results from studies describing the biodistribution of stem cells transplanted in vivo have been consistent on this point. After systemic administration of stem cells, despite early accumulation at the site of injury, few cells might permanently engraft within the injured kidney; most of the cells are trapped in the liver, lungs, and spleen [2, 4, 46]. Due to their smaller size, SC-EVs can easily pass through the blood–tissue barrier to the injured site. It has been demonstrated that as early as 1 h after intravenous injection, PKH26-labeled SC-EVs can be detected in the kidneys of mice with ischemia/reperfusion (I/R)-induced AKI. Six hours later, even in tubular epithelial cells, transplanted SC-EVs can be observed [47]. The specific localization and more extensive biodistribution of SC-EVs in injured renal tissues may be sufficient to promote regenerative events.

The capture of SC-EVs by injured renal cells depends on bidirectional communication between renal cells and SC-EVs. SC-EVs express several adhesion molecules for internalization, such as CD44 and CD29. In vitro, preincubation of SC-EVs with specific blocking antibodies diminishes the internalization of labeled SC-EVs within proximal tubular epithelial cells (PTECs) [48]. In vivo, the failure of trypsin-treated SC-EVs to enter the kidneys of mice with AKI has also confirmed the requirement of surface molecules for SC-EV entrance into target cells [13]. Signals from injured renal cells are also instrumental for the incorporation of SC-EVs, based on the finding that no intravenously injected SC-EVs can be detected within kidneys under physiological conditions [49].

Captured SC-EVs can subsequently transfer their biologically active contents to injured renal cells and further promote tissue regeneration via genetic communication. The mechanism will be discussed in the following section.

## The regenerative effect of stem cells in AKI is dependent on genetic communication by EVs

**Fig. 2:**
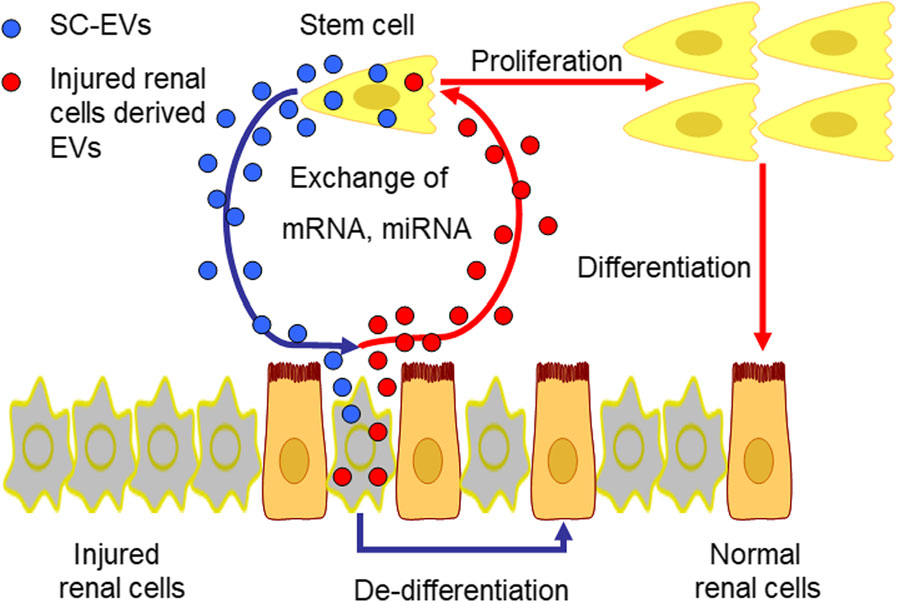
Importance of EVs as vehicles for genetic communication between stem cells and injured renal cells during AKI. By delivering mRNAs and miRNAs, SC-EVs may activate regenerative programs in injured renal cells and stimulate them to dedifferentiate into normal renal cells. Alternatively, EVs originating from injured renal cells also contain genetic information that is believed to be able to influence the phenotypic and functional changes of stem cells, favoring renal recovery. SC-EVs, stem cells derived EVs; EVs, extracellular vesicles

The idea that the regenerative effect of stem cells in AKI is based on their paracrine/endocrine activity has been accepted by most experts. However, the concept that genetic communication exists between stem cells and injured renal cells arose only a decade ago (Fig. 2). Above, we mentioned that SC-EVs are considered vehicles that can transfer genetic information and change the phenotypes and functions of target cells. In the context of renal regeneration, it is plausible that EVs can participate in genetic communication, transfer genetic information to target cells, alter target gene regulatory networks, and accelerate renal recovery. The possibility of this mechanism provides new insights to support the paracrine hypothesis of stem cell-based therapy in AKI.

### Evidences suggesting the existence of genetic information transfer from SC-EVs to injured renal cells in AKI

Although horizontal transfer of genetic molecules from SC-EVs to injured renal cells in AKI has not been observed, the studies described in this section have hinted at the existence of this mechanism with the help of RNase, drosha/dicer-knockdown, specific antagomirs, and gene ontology analysis (Table 1).

**Table 1:** Evidence suggesting the existence of genetic information transfer from SC-EVs to injured renal cells in AKI

References	Year	Sample	AKI model	EVs type	Stem cells source	Outcomes
Gatti [50]	2011	Rats	I/R	MVs	MSCs	↑Cell proliferation, ↓apoptosis, ↑renal function; pretreatment of MVs with RNase abrogated these protective effects.
Ranghino [47]	2017	Mice	I/R	EVs	MSCs	↑Cell proliferation, ↑renal function; RNase-inactivated EVs were ineffective.
Zou [53]	2014	Rats	I/R	MVs	WJMSCs	↑Cell proliferation, ↓apoptosis, ↑renal function, ↓CX3CL1, ↓macrophages infiltration, ↓α-SMA; the downregulated expression of CX3CL1 was related with miRNAs in WJMSC-MVs (miR-15a, miR-15b, and miR-16)
Cantaluppi [54]	2012	Rats	I/R	MVs	EPCs	↑Cell proliferation and angiogenesis, ↓Apoptosis, ↑renal function, ↓leukocyte infiltration; all of the protective effects was inhibited after RNase or specific antagomiRs, anti-miR-126 and anti-miR-296 treatment
Reis [51]	2012	Rats	Gentamicin	Exosomes	MSCs	↑Cell proliferation, ↓apoptosis, ↑renal function; the protective effects were blunted after treated with RNase
Collino [52]	2015	Mice	Glycerol	EVs	MSCs	↑Genes acting in metabolic pathways, ↓genes involved in inflammation, cell adhesion molecules, and cell cycle, ↓Lcn2, ↓fibrinogen β, ↑renal function; knockdown of Drosha for downregulation of miRNAs in EVs reduced their kidney proregenerative properties

*SC-EVs* stem cell-derived extracellular vesicles, *AKI* acute kidney injury, *EVs* extracellular vesicles, *I/R* ischemia/reperfusion, *MVs* microvesicles, *MSCs* mesenchymal stem cells, *WJMSCs* Wharton’s Jelly mesenchymal stromal cells, *EPCs* endothelial progenitor cells

Among the many different types of SC-EVs, EVs originating from mesenchymal stem cells (MSC-EVs) were the first to be shown to be able to transfer genetic information in preclinical AKI models. A single administration of MSC-EVs immediately after renal I/R injury protected rats from AKI by stimulating cell proliferation and inhibiting apoptosis. Preincubation of MSC-EVs with RNase, an inactivator targeting RNA in the cargoes of MSC-EVs, abolished these protective effects, indicating that transfer of RNA-like molecules by MSC-EVs might account for their therapeutic effect [50]. Similar results were also obtained by Ranghino et al. [47] and Reis et al. [51] in either I/R- or gentamicin-induced AKI models. Drosha is an enzyme responsible for the cleavage of inactive pri-miRNA into precursor miRNA and is an excellent tool for miRNA investigation. Depletion of drosha in MSC-EVs leads to global downregulation of miRNAs. These alterations in miRNA levels reverse the morphologic and functional recovery of AKI mediated by MSC-EVs as donor EVs. Gene ontology analysis has demonstrated that these downregulated miRNAs are key factors in restoring the function of a variety of disorganized genes associated with fatty acid metabolism, inflammation, matrix-receptor interaction, and cell adhesion molecules during AKI [52].

In addition to evidence from studies using nonspecific RNA degradation methods, there also exists some evidence indicating that specific kinds of RNAs shuttled by SC-EVs are transferred and contribute to the regenerative potential of SC-EVs. Injected Wharton’s jelly-derived mesenchymal stem cell-derived EVs (WJMSC-EVs) have been found to induce decreases in the expression of CX3CL1, further lessening the infiltration of macrophages in I/R-injured kidneys. To further investigate the participation of WJMSC-EVs in the process of genetic information transfer, the authors matched the miRNAs that were predicted to target CX3CL1 in the TargetScan database with the highly expressed miRNAs shuttled by WJMSC-EVs. Ultimately, they found that miR-16, miR-15b, and miR-15a might transfer from WJMSC-EVs to injured renal cells, modulate the expression of CX3CL1, and ameliorate renal injury [53]. Similarly, transfection with selective miR antagomirs to deplete proangiogenic miR-126 and miR-296 in endothelial progenitor cell-derived EVs (EPC-EVs) has been found to inhibit the protective effects of EVs in an I/R-induced AKI model [54].

### Evidence demonstrating the existence of horizontal mRNA transfer from SC-EVs to injured renal cells in AKI

Based on the results mentioned above, it remains difficult to state that there exists horizontal transfer of RNA from SC-EVs to injured renal cells in AKI. The RNA variations in SC-EV-treated renal cells could be due to transcriptional effects mediated by the renal cells themselves rather than by direct delivery from SC-EVs. Distinguishing the origins of the RNAs and verifying their biological effects may help to address this uncertainty (Table 2).

**Table 2:** Evidence demonstrating the existence of horizontal mRNA transfer from SC-EVs to injured renal cells in AKI

References	Year	Sample	AKI model	EVs type	Stem cells source	Outcomes
Bruno [13]	2009	Mice	Glycerol	MVs	MSCs	↑Transfer of mRNA, ↑cell proliferation, ↓apoptosis, ↑renal function
Bruno [55]	2012	Mice	Cisplatin	MVs	MSCs	↑Transfer of mRNA, ↓apoptosis, ↑renal function
Tomasoni [56]	2013	PTECs	Cisplatin	Exosomes	MSCs	↑Transfer of IGF-1R mRNA, ↑cell proliferation
Ju [57]	2015	Rats	I/R	MVs	MSCs	↑Transfer of HGF mRNA, ↑HGF, ↓α-SMA, ↑cell proliferation and dedifferentiation, ↓apoptosis, ↑renal function

*SC-EVs* stem cell-derived extracellular vesicles, *AKI* acute kidney injury, *EVs* extracellular vesicles, *MVs* microvesicles, *MSCs* mesenchymal stem cells, *PTECs* proximal tubular epithelial cells, *IGF-1R* insulin-like growth factor-1 receptor, *I/R* ischemia/reperfusion, *HGF* hepatocyte growth factor

After transplanting MSC-EVs in a glycerol-induced AKI model, Bruno et al. obtained results consistent with those of Gatti et al. To determine whether there existed horizontal transfer of genetic information from MSC-EVs to injured renal cells, the researchers labeled MSC-EVs with the human genes POLR2E and SUMO-1. RT-PCR confirmed that the mRNA of the human gene POLR2E was present in the kidneys of mice treated with MSC-EVs after AKI, indicating that this mRNA was horizontally transferred from MSC-EVs. Moreover, accumulation of related proteins was also found with nuclear localization in the tubules of AKI mice, suggesting that the specific mRNA shuttled by MSC-EVs could further be translated into target proteins in vivo [13]. In 2012, the researchers again confirmed this phenomenon in a lethal model of cisplatin-induced AKI in SCID mice [55]. To confirm whether biologically active mRNAs could be transferred by SC-EVs to injured renal cells, Tomasoni et al. incubated human MSC-EVs that contained insulin-like growth factor-1 receptor (IGF-1R) with R- mouse fibroblast cell lines that lacked IGF-1R. RT-PCR analysis revealed that the IGF-1R transcript could be detected in incubated R- cells, indicating the delivery of mRNAs from human MSC-EVs. The translated protein was also found in incubated R- cells but not in human MSC-EVs, indicating the biological activity of the transferred mRNAs. Coincubation of IGF-1R-silenced MSC-EVs with cisplatin-pretreated PTECs abolished the beneficial effect, suggesting that the transferred mRNAs play a fundamental role in renal regeneration [56]. Similarly, after injection of human MSC-EVs into rats suffering from I/R-induced AKI, human hepatocyte growth factor (HGF) mRNA and corresponding proteins appeared in rat tubular cells and facilitated renal cell dedifferentiation and growth. All these findings indicate that the delivery of functional mRNAs from SC-EVs to injured renal cells could also occur in in vivo AKI models [57].

### Evidence demonstrating the existence of horizontal transfer of miRNA from SC-EVs to injured renal cells in AKI

miRNAs are a class of single-stranded small RNAs approximately 19–24 nucleotides in length that participate in the posttranscriptional modulation of gene expression and are involved in the regulation of several cellular physiological activities [58]. AKI induces significant miRNA chaos within renal cells. Horizontal transfer of some cell repair-associated miRNAs from SC-EVs to injured renal cells may account for the protective effect of SC-EVs in AKI (Table 3).

**Table 3:** Evidence demonstrating the existence of horizontal transfer of miRNA from SC-EVs to injured renal cells in AKI

References	Year	Sample	AKI model	EVs type	Stem cells source	Outcomes
Lindoso [48]	2014	PTECs	I/R	EVs	MSCs	↑Transfer of miR-148b-3p, miR-410, miR-495, and miR-548c-5p, ↑cell viability
Vinas [61]	2016	Mice	I/R	Exosomes	ECFCs	↑Transfer of miR-486-5p, ↓PTEN, ↑Akt phosphorylation, ↓apoptosis, ↑renal function
Gu [62]	2016	Rats	I/R	EVs	WJMSCs	↑Transfer of miR-30, ↓DRP-1, ↓mitochondrial fragmentation, ↓apoptosis, ↑renal function

*SC-EVs* stem cell-derived extracellular vesicles, *AKI* acute kidney injury, *EVs* extracellular vesicles, *PTECs* proximal tubular epithelial cells, *I/R* ischemia/reperfusion, *MSCs* mesenchymal stem cells, *ECFCs* endothelial colony-forming cells, *PTEN* phosphatase and tensin homolog, *WJMSCs* Wharton’s Jelly mesenchymal stromal cells, *DRP-1* dynamin-related protein-1

Although not established in an AKI microenvironment, tubular epithelial cells cocultured with MSC-EVs showed an abundance of some specific fluorescently labeled miRNAs carried by MSC-EVs in one study. These miRNAs included miR-21, miR-100, miR-99a, and miR-223, which target phosphatase and tensin homolog (PTEN), cyclin D1, and Bcl-2, suggesting a mechanism of miRNA delivery [59]. Data obtained by Zou et al. demonstrated that administration of MSC-EVs into a rat I/R-induced AKI model decreased the infiltration of NK cells and ameliorated renal injury. In in vitro studies, MSC-EVs have been labeled with both SYTO (an RNA label) and PKH-26 (a cytomembrane label). After incubation with these double-labeled MSC-EVs, both tubular epithelial cells (TECs) and human umbilical vein endothelial cells (HUVECs) showed the incorporation of MSC-EVs and the transfer of RNAs. MiRNA array analysis revealed a total of 25 upregulated miRNAs in MSC-EVs compared with fibroblast cell-derived EVs, suggesting that the upregulated miRNAs might constitute the transferred cargoes [60]. The first evidence demonstrating the existence of horizontal transfer of miRNA in the context of SC-EV therapy during AKI was obtained by Lindoso et al. These authors cocultured labeled MSC-EVs with PTECs and observed the incorporation of MSC-EVs into the cells by confocal microscopy. Subsequently, ATP depletion injury of the PTECs was induced to mimic I/R-induced AKI. After 24 h of incubation, a 2.7-fold increase in the uptake of MSC-EVs by PTECs was confirmed by FACS analysis. Given the theory that internalization of MSC-EVs might lead to the transfer of MSC-EV cargoes, the authors then evaluated the miRNA content inside PTECs treated with MSC-EVs. Thirteen types of miRNAs were upregulated in the PTECs. Experiments performed in the presence of a transcription inhibitor ultimately demonstrated that the miRNAs miR-148b-3p, miR-410, miR-495, and miR-548c-5p were horizontally transferred from MSC-EVs [48]. In vivo evidence was obtained in a study conducted by Vinas et al. in 2016. After transplantation of endothelial colony-forming cell-derived EVs (ECFC-EVs), which were enriched for miR-486-5p, increased kidney miR-486-5p levels together with reduced apoptosis and improved renal function were observed. Infusion of miR-486-5p antagomir-pretreated ECFC-EVs did not induce a protective effect, indicating that the underlying beneficial effect of ECFC-EVs was due to the transfer of miR-486-5p. In vitro, the application of GW4869 (an inhibitor of exosome secretion) or EIPA (an inhibitor of exosome uptake) blocked the increase in miR-486-5p levels in recipient cells. All of these results verified the existence of miR-486-5p horizontal transfer [61]. Similarly, the transfer of miR-30 from WJMSC-EVs has also been identified by Gu et al. Elevated levels of exogenous miR-30 in renal tubular cells could relieve the activation of dynamin-related protein-1 (DRP-1) and mitochondrial fragmentation induced by I/R-induced AKI, which led to antiapoptotic and renoprotective effects [62].

### Evidences suggesting the existence of genetic information transfer from injured renal cell-derived EVs to stem cells in AKI

Above, we discussed how SC-EVs confer upon injured cells a self-regenerative ability through cell–cell communication. The recently proposed continuum model of stem cell biology supports the possibility that tissue-specific information could also be transferred from injured cells to stem cells by EVs. This transfer could influence the phenotypic and functional changes of stem cells. Therefore, there may exist a reciprocal exchange of information between stem cells and injured cells.

Incubation of bone marrow cells with injured lung cells induces epigenetic modifications in the bone marrow cells, with evidence that some lung-specific proteins, such as surfactant B and Clara cell-specific protein, are expressed [63]. Expression of prostate, brain, and liver mRNAs has also been found in marrow cells after coculture with specific tissues [64, 65]. Stem cell plasticity is a phenomenon in which stem cells located in specific niches are able to differentiate into target cells based on communication with neighboring cells [66]. Importantly, the signals transferred between the microenvironment and stem cells may rely on EVs [67]. Chiabotto et al. found that after incubation with PTECs, MSCs could uptake EVs released from PTECs that contained large amounts of miRNAs belonging to the miR-200 family. The elevated expression of these miRNAs in MSCs was the main mediator of a mesenchymal-epithelial transition that laid the foundation for their regenerative potential in AKI [68]. EVs secreted by injured or degenerated tissues stimulate stem cell reentry into the cell cycle, trigger terminal stem cell differentiation, and participate in the physiological process of tissue repair [69, 70].

Can injured renal cell-derived EVs transfer genetic information to stem cells during AKI, provide inductive signals for proliferation and differentiation, and accelerate renal recovery? Unlike in many other injured organs, transplanted stem cell engraftment into injured kidneys and subsequent proliferation and differentiation into normal renal cells are rare. This fact makes it difficult to believe the abovementioned hypothesis could be valid. However, several studies have reported the existence of a resident population of stem cells within nephrons [71, 72]. Moreover, it has also been recently demonstrated that EPCs could be recruited into the kidneys after AKI [73, 74]. All of these findings indicate that the hypothesis described above is indeed valid.

To further test this hypothesis, Baer et al. prepared a conditioned medium from injured PTECs and transferred it to MSC cultures. After incubation in this medium, significant proliferation of MSCs and phosphorylation of ERK1/ERK2 were observed. Moreover, both cell morphology observation and surface marker analysis suggested that the MSCs underwent a phenotypic change to become epithelial-like cells. These findings strongly indicated that signals from injured renal cells could induce the proliferation and differentiation of stem cells [75]. The results from another study showed that mice overexpressing miR-126 in the hematopoietic compartment were more resistant to I/R-induced AKI. Overexpression of miR-126 in the hematopoietic compartment could recruit hematopoietic stem and progenitor cells toward the injured kidneys and protect kidneys against AKI via preservation of microvascular integrity [76]. AKI also induces the upregulation of miR-126 in renal cells [77]. Whether this miRNA can be transferred by injured renal cell-derived EVs to stem cells and in return accelerate renal recovery is a question deserving further research.

## Conclusions and future challenges

The existing evidence suggests that genetic communication by EVs may be an important mechanism underlying stem cell-based therapy-mediated protection against AKI. SC-EVs are emerging as potent genetic information sources that deliver mRNAs and miRNAs to injured renal cells and exert renoprotective effects. On the other hand, EVs originating from injured renal cells also contain genetic information that is believed to be able to influence the phenotypic and functional changes of stem cells, favoring renal recovery.

Despite the promising future of SC-EV therapy in AKI management, some challenges should still be addressed before its clinical application. First, there remains a lack a consensus on the definition of SC-EVs. Different fractions of SC-EVs may have different functions [78]. For instance, a study by Aliotta et al. reported that exosomes, but not MVs, derived from MSCs were effective in treating monocrotaline-induced pulmonary hypertension [79]. In contrast, total EVs containing both exosomes and MVs derived from MSCs show a better regenerative effect in reversing radiation damage than either MVs or exosomes alone [80]. Developing a robust definition of SC-EVs and a reproducible method for SC-EV isolation and establishing a standard SC-EV regimen is of great importance. Second, the mechanisms underlying the selective sorting of genetic information into SC-EVs are far from being understood. Since current techniques for gene transfer are still confined to viral or synthetic agents, engineered EVs expressing specific mRNAs, miRNAs, and other materials may be promising for delivering targeted genetic information and avoiding potential safety problems. Third, although our article concludes that genetic communication is an important mechanism underlying SC-EV-mediated protection against AKI, the mechanisms of the beneficial effects of SC-EVs in AKI are far from established. Clarifying the detailed renoprotective mechanism may expand the success of regenerative medicine in AKI patients. Finally, problems related to immunological rejection and tumorigenicity should always be considered.

We look forward to a bright future of stem cell-based therapy in AKI and call for more research in this area.

## Abbreviations

AKI: Acute kidney injury
Alix: Apoptosis-linked gene-2-interacting protein x
APCs: Antigen-presenting cells
DRP-1: Dynamin-related protein-1
ECFC-EVs: Endothelial colony-forming cell-derived EVs
EPC-EVs: Endothelial progenitor cell-derived EVs
EVs: Extracellular vesicles
HGF: Hepatocyte growth factor
HSP: Heat shock protein
HUVECs: Human umbilical vein endothelial cells
I/R: Ischemia/reperfusion
IGF-1R: Insulin-like growth factor-1 receptor
MHC: Major histocompatibility complex
MSC-EVs: EVs originated from mesenchymal stem cells
MVs: Microvesicles
PTECs: Proximal tubular epithelial cells
PTEN: Phosphatase and tensin homolog
SC-EVs: Stem cell-derived EVs
TECs: Tubular epithelial cells
WJMSC-EVs: Wharton-Jelly mesenchymal stem cell-derived EVs
